# How does a family history of psychosis influence the risk of methamphetamine‐related psychotic symptoms: Evidence from longitudinal panel data

**DOI:** 10.1111/add.16230

**Published:** 2023-05-30

**Authors:** Rebecca McKetin, Philip J. Clare, David Castle, Alyna Turner, Peter J. Kelly, Dan I. Lubman, Shalini Arunogiri, Victoria Manning, Michael Berk

**Affiliations:** ^1^ National Drug and Alcohol Research Centre University of New South Wales Sydney Australia; ^2^ Prevention Research Collaboration, School of Public Health University of Sydney Camperdown Australia; ^3^ Charles Perkins Centre University of Sydney Camperdown Australia; ^4^ Centre for Addiction and Mental Health and Department of Psychiatry University of Toronto Toronto Canada; ^5^ Deakin University, IMPACT Institute for Innovation in Physical and Mental Health and Clinical Translation School of Medicine Geelong Australia; ^6^ School of Psychology and Illawarra Health and Medical Research Institute University of Wollongong Wollongong Australia; ^7^ Monash Addiction Research Centre, Eastern Health Clinical School, Faculty of Medicine, Nursing and Health Sciences Monash University Melbourne Australia; ^8^ Turning Point, Eastern Health Richmond Australia; ^9^ Orygen, The National Centre of Excellence in Youth Mental Health, the Department of Psychiatry, and the Florey Institute of Neuroscience and Mental Health The University of Melbourne Parkville Australia

**Keywords:** mental disorders, methamphetamine, psychiatry, psychosis, risk factors, schizophrenia, substance use

## Abstract

**Aims:**

To determine whether the risk of psychotic symptoms during weeks of methamphetamine use was dependent on, increased by, or independent of having a family history of psychosis.

**Design:**

Secondary analysis of 13 contiguous 1‐week periods of data (1370 weeks). A risk modification framework was used to test each scenario.

**Setting:**

Geelong, Wollongong and Melbourne, Australia.

**Participants:**

Participants in a randomized controlled trial of treatment for methamphetamine dependence (*n* = 148) who did not have a primary psychotic disorder on enrolment.

**Measurements:**

Psychotic symptoms in the previous week were defined as a score of 3+ on any of the Brief Psychiatric Rating Scale items of hallucinations, unusual thought content or suspiciousness. Any (vs no) methamphetamine use in the previous week was assessed using the Timeline Followback method. Self‐reported family history of psychosis was assessed using the Diagnostic Interview for Psychosis.

**Findings:**

The risk of psychotic symptoms in the past week was independently associated with methamphetamine use in that week (relative risk [RR] = 2.3, 95% CI = 1.3–4.3) and with having a family history of psychosis (RR = 2.4, 95% CI = 0.9–7.0); the joint risk among participants with a family history of psychosis during weeks when they were using methamphetamine was large (RR = 4.0, 95% CI = 2.0–7.9). There was no significant interaction between a family history of psychosis and methamphetamine use in predicting psychotic symptoms (interaction RR = 0.7 95% CI = 0.3–1.8), but there was a small non‐significant excess risk due to the interaction (0.20 95% CI = −1.63 to 2.03).

**Conclusions:**

Among people dependent on methamphetamine, the relative risk of psychotic symptoms during weeks of methamphetamine use does not appear to be dependent on, or increased by, having a family history of psychosis. However, a family history of psychosis does appear to be an independent risk factor that contributes to the absolute risk of psychotic symptoms in this population.

## INTRODUCTION

Methamphetamine use disorder affects an estimated 7.4 million people worldwide (95% CI = 5.4–9.8 million) [1]. Psychosis is a significant public health concern attached to the drug's use [2]. Repeated intoxication with high dose methamphetamine use can incite a transient schizophreniform psychosis [3], the most common symptoms of which are paranoia and hallucinations [4]. This psychosis typically lasts hours to days and recedes once the drug has been eliminated from the body [5, 6]. More frequent methamphetamine use and more severe dependence are the most consistently observed risk factors [7], with the risk of psychotic symptoms increasing in a dose‐related fashion with more days of use [3]. Although there is considerable variation in vulnerability to methamphetamine‐related psychosis, other potential risk factors (including other methamphetamine use patterns) have either not been robustly examined or have not been consistently observed [7].

Given the high heritability of psychotic experiences [8], one likely risk factor is having a family history of psychosis. In support of this, familial morbidity for schizophrenia has been found to significantly increase the risk of methamphetamine‐related psychosis [9, 10] and substance‐induced psychoses more generally [11]. Methamphetamine use also increases the risk of onset schizophrenia [12] and can precipitate and exacerbate psychotic symptoms in people with the disorder [13]. This evidence suggests that methamphetamine use may be precipitating psychotic symptoms in people who have a genetic vulnerability [14]. However, genetic studies have found that although familial morbidity plays a role in the risk of developing drug‐induced psychoses, substantial drug exposure is also a requirement [11]. Hence, it is likely that both methamphetamine and a family history of psychosis are risk factors for psychosis, possibly acting in a synergistic fashion to increase the risk of psychotic symptoms.

We sought to better understand how having family history of psychosis influences the risk of psychotic symptoms among people dependent on methamphetamine. We used longitudinal panel data (13 weeks of continuous data) to examine whether having a family history of psychosis modified the risk of psychotic symptoms occurring during weeks of methamphetamine use. Participants in this study were volunteering for a clinical trial and had no known personal history of either schizophrenia or other primary psychoses. We used a risk modification framework [15] to explore how our data fitted with three possible scenarios and to consider the impact of family history on the relative and absolute risk of psychosis in the population (see Saracci *et al*. for an example of this approach) [16].

(1) Scenario 1: The risk of methamphetamine‐related psychosis is dependent on having a family history of psychosis. In this scenario, there should be an increase in the relative risk (RR) of psychotic symptoms during weeks of methamphetamine use, but only for people with a family history of psychosis (not for people without a family history of psychosis).
(2) Scenario 2: Methamphetamine use and a family history of psychosis are independent risk factors for psychosis. In this scenario, there should be a similar increase in the RR of psychotic symptoms during weeks of methamphetamine use for people with and without a family history of psychosis. The joint risk should be the addition of the risk associated with methamphetamine use and a family history of psychosis.
(3) Scenario 3: Both methamphetamine use and a family history of psychosis increase the risk of psychosis (as in scenario 2), but having a family history of psychosis further increases vulnerability to methamphetamine‐related psychotic symptoms. In this scenario, the RR of psychotic symptoms during weeks of methamphetamine use should be greater in people with a family history of psychosis compared to people with no family history of psychosis. The joint risk of both methamphetamine use and having a family history of psychosis should exceed the risk associated with both factors independently.

## METHODS

### Participants and procedures

This is a secondary analysis of data from a randomized controlled trial of *N*‐Acetylcysteine for methamphetamine use [17, 18]. Participants were self‐selecting and identified as wanting to reduce their methamphetamine use. Details the protocol and ethics approvals can be found at the trial registration (ACTRN12618000366257; see http://www.anzctr.org.au/). Guidelines for Strengthening the Reporting of Observational Studies in Epidemiology (STROBE) [19] were followed (see Supporting Information for the STROBE Checklist).

Data collection took place between July 2018 and March 2020. All participants provided written informed consent before study entry and were reimbursed AU$30 per assessment. Recruitment took place via collaboration with local service providers (e.g. needle and syringe programs), media, Facebook advertisements, a dedicated website, flyers and word‐of‐mouth. Inclusion criteria were being ages 18 to 60 years, being dependent on methamphetamine, seeking to reduce methamphetamine use and being able to provide informed consent and comply with the trial protocol. People were excluded from participation if they were enrolled in other substance use treatment at the time of enrolment (including pharmacotherapy for substance use disorders), had a primary psychotic disorder, were in need of acute care for psychiatric or other major medical conditions, had a positive pregnancy screen at baseline (or were not able to avoid pregnancy during the trial), or had contraindications for taking *N*‐Acetylcysteine.

Participants included in this analysis (*n* = 148) were dependent on methamphetamine and did not have a prior primary psychotic disorder. Diagnostic and Statistical Manual of Mental Disorders (DSM)‐IV methamphetamine dependence was confirmed using the Composite International Diagnostic Interview Version 3.0 [20]. Participants were screened for a primary psychotic disorder using the Mini International Neuropsychiatric Interview (MINI) for Schizophrenia and Psychotic Disorders Studies, English Version 7.0.1 [21], and the diagnosis was confirmed by the trial physician. Data were taken from the trial baseline assessment and the 12 subsequent weekly assessments (13 assessments in total). Of the 153 participants randomized in the trial, four were excluded from the current analysis because they were lost to follow‐up and therefore, had no data on psychotic symptoms. One remaining participant did not know their family history of psychosis because of adoption, giving our sample of 148 participants. A total of 69% of the next 12 assessments were completed. Data on psychosis were missing from an additional nine assessments, giving 1359 data points for analysis.

### Measures

#### Time‐varying measures

Time‐varying measures were assessed at baseline and each week throughout the 12‐week follow‐up.

##### Psychotic symptoms

Psychotic symptoms were assessed for the past week at each weekly assessment. Active psychotic symptoms were defined as a score of three or more on any of the Brief Psychiatric Rating Scale (BPRS) [22] items of suspiciousness, unusual thought content or hallucinations at any time during that week. These three BPRS items load on a common factor related to positive psychotic symptoms associated with methamphetamine use [23, 24]. A cut‐point of three on the BPRS items reflects at least mild psychotic symptoms. Inter‐rater reliability on 161 audiotaped BPRS ratings gave an inter‐rater agreement of 83% (κ = 0.51) for this definition of psychotic symptoms [18].

##### Substance use

Self‐reported days of methamphetamine use in the 7 days before each assessment were obtained using the Timeline Followback (TLFB) method [25]. There was good concordance between self‐reported methamphetamine use in the past week and methamphetamine positive oral fluid tests [26]. Days of use in the previous week were assessed for major drug classes (tobacco, alcohol, cannabis, cocaine, ecstasy, hallucinogens, inhalants, heroin and other opioids). Concomitant medications were monitored at each weekly assessment and coded according to classifications used in the Australian medical index (Monthly Index of Medical Specialities) [27].

#### Time‐invariant measures

All time‐invariant measures were taken at baseline.

##### Family history of psychosis

Each participant's family history of psychosis was assessed using questions from the Diagnostic Interview for Psychosis (DIP) [28]. A family history of psychosis included having a relative (first, second or third‐degree relative) with either schizophrenia or bipolar disorder (or other affective disorder involving psychosis). It excluded a family history of substance‐induced psychosis.

##### Sociodemographics

Demographics included sex (male vs female), age and indicators of socio‐economic status: net income in the previous fortnight (<AU$200, $200–399, $400–799, $800–1199, $1200 or more); education (years of schooling; completion of a technical or university qualification) and employment status (unemployed, casual/part‐time, full‐time, student or home duties).

##### Methamphetamine use history

Methamphetamine use history included duration of use (in years), age of first methamphetamine use (in years) and primary route of administration at baseline (injecting vs other).

### Analyses

This analysis was not preregistered and should be considered exploratory. The analyses were conducted in Stata Version 17.0 (Statacorp). All tests were two‐sided with significance set at *P* < 0.05 (with 95% CIs reported). For descriptive data, comparisons were made using Pearson's χ^2^ tests for categorical data, *t*‐tests for normally distributed continuous data and median comparison tests for highly skewed data, where medians and interquartile ranges (IQRs) are presented. Inferences about small, medium and large effect sizes were based on Olivier *et al*. [29].

We applied modified Poisson random effects models [30] to panel data to estimate the RR of psychotic symptoms in the past week by methamphetamine use in that week. These population averaged models were implemented using the Stata panel data command suite to include a random intercept term to account for clustering of data on repeated measures. We used robust error estimates (Huber‐White Sandwich estimator) and an exchangeable correlation matrix. This approach has been validated for estimating risk in clustered prospective data [31] and is robust to small cell sizes [30].

The outcome in all models was 13 repeated time‐varying assessments of psychotic symptoms in the previous week. The predictor was time‐varying use of methamphetamine in the previous week (no use [0] vs any use [1]). A family history of a psychotic disorder (either schizophrenia or bipolar disorder) was the modifier. Confounders were any variables measured in the study that were putative risk factors for psychosis: younger age [32], being male [32], socio‐economic deprivation [33], early onset methamphetamine use [34, 35], longer duration methamphetamine use [36] and other drug use [3, 34]. Indicators of socio‐economic deprivation included in the analysis were net income in the previous fortnight; years of schooling and unemployment (vs employed, student or home duties). Other drug use measures were time‐varying and included past week use of tobacco, alcohol and cannabis use and a variable ‘other drug use’ representing whether or not the participant used any other drug class.

Our examination of effect modification was based on the principles recommended by Knol and Vanderweele [37]. The risk of psychotic symptoms by methamphetamine use (i.e. any past week use vs no use in the past week) was derived for each level of the risk factor (i.e. participants with family history of psychosis and participants without a family history of psychosis). Risk ratios were also derived for each risk quadrant using a factorial contrast relative to no methamphetamine use and no family history of psychosis (i.e. family history of psychosis + no past week methamphetamine use [1]; no family history of psychosis + past week methamphetamine use [2]; family history of psychosis + past week methamphetamine use [3], relative to no family history of psychosis + no past week methamphetamine use [0]). Effect modification on the multiplicative (risk ratio) scale was estimated using an interaction contrast. The relative excess risk due to the interaction (RERI) and the attributable proportion (AP) of psychosis cases due to the interaction and 95% CIs were calculated using the methods presented in Hosmer and Lemeshow [38]. Models were adjusted for covariates that were associated with either past week methamphetamine use or past week psychotic symptoms.

Four sets of sensitivity analyses were undertaken:

(1) Replacing any versus no use of methamphetamine in the past week with a continuous variable of days of methamphetamine use in the past week.
(2) Missing data were imputed in R (Version 4.0.3) using chained equations (see Section 5 of the Supporting Information for detail; information on missing data can be found in Table S1 and Figure S1).
(3) Data affected by antipsychotic use were removed from the analysis (194 weeks of data from 19 participants).
(4) The outcome variable of any versus no psychotic symptoms in the past week was replaced with a variable representing the severity of psychotic symptoms in the past week (see Section 7.4 of the Supporting Information for details).

## RESULTS

### Participant characteristics

Participants had a mean age of 38 (SD = 8) years and 60% were male. They had used methamphetamine on a median (IQR) of 24 (16–28) days in the 4 weeks before baseline. Thirty‐five per cent injected methamphetamine (64% smoked; 1% used other non‐parenteral routes). Other substance use consisted primarily of tobacco, alcohol and cannabis (Table 1). Twenty‐seven participants (18% of the sample) reported a family history of a psychotic disorder (10 for bipolar disorder, 14 for schizophrenia and three for both). Of these, 48% involved a first‐degree relative (30% second‐degree; 19% third degree).

**TABLE 1:** Correlates of psychotic symptoms in the past week.

Time‐invariant baseline variables:	Per participant (*n* = 148)	RR (95% CI)	*P*‐value
Family history of psychosis, *n* (%)	27 (18)	1.72 (1.22–2.43)	0.002
Male, *n* (%)	89 (60)	1.01 (0.71–1.44)	0.948
Age, mean (SD)	38 (8)	0.98 (0.97–1.00)	0.098
Years of schooling, median (IQR)	11 (10–12)	1.08 (0.97–1.20)	0.180
Unemployed, *n* (%)	82 (55)	1.03 (0.73–1.46)	0.858
Income^a^, *n* (%)			
<400	20 (14)	Reference	
400–799	71 (48)	1.09 (0.64–1.83)	0.754
800–1199	23 (16)	0.70 (0.36–1.34)	0.283
1200+	34 (23)	0.91 (0.49–1.70)	0.773
Age of first methamphetamine use, mean (SD) years	22 (8)	1.00 (0.98–1.02)	0.744
Duration of methamphetamine use, mean (SD) years	15 (9)	0.99 (0.97–1.01)	0.190
Injecting methamphetamine, *n* (%)	52 (35)	1.07 (0.76–1.50)	0.712

Time‐varying drug use variables:	Per week (*n* = 1359)	RR (95% CI)	
Any use in the past week, *n* (%)			
Methamphetamine	1150 (85)	2.05 (1.27–3.32)	0.003
Cannabis	496 (37)	1.23 (0.92–1.67)	0.160
Alcohol	565 (42)	1.06 (0.87–1.29)	0.546
Tobacco	1108 (82)	1.06 (0.75–1.50)	0.751
Any other drug^b^	181 (13)	1.29 (0.98–1.70)	0.072
Days of use in the past week, mean (SD)			
Methamphetamine	5 (3)	1.11 (1.06–1.17)	<0.001
Cannabis	2 (3)	1.04 (0.98–1.09)	0.183
Alcohol	1 (2)	1.00 (0.95–1.06)	0.912
Tobacco	6 (3)	1.01 (0.96–1.07)	0.742

Abbreviations: IQR, interquartile range; RR, relative risk.

^a^ AU$ net per fortnight.

^b^ Use of any other drug class (cocaine, ecstasy, hallucinogens, inhalants, heroin or other opioids).

### Methamphetamine use and psychotic symptoms during the study period

Methamphetamine was used during 85% of weeks, on a median of 5 days (IQR = 3–7). Psychotic symptoms occurred during 26% of weeks, with 64% of participants having a psychotic symptom at one or more assessments (median = 3 assessments, IQR = 2–6). Psychotic symptoms in the past week were associated with methamphetamine use in the past week (both any use in the past week and days of use in the past week) and having a family history of psychosis, but not other covariates (Table 1).

### Effect modification analysis

Table 2 shows the various effect modification analyses. These have been adjusted for covariates that were associated with either past week psychotic symptoms (Table 1) or past week methamphetamine use (see Table S2). Unadjusted analyses can be found in Table S3.

**TABLE 2:** Effect modification analysis for family history of psychosis on the risk of psychotic symptoms during weeks of methamphetamine use.

	No past week methamphetamine use			Past week methamphetamine use			Within strata effect		Interaction effect		Relative excess risk due to the interaction^a^	Attributable proportion of cases^b^
	Past week psychotic symptoms	RR (95% CI)	*P*‐value	Past week psychotic symptoms	RR (95% CI)	*P*‐value	RR (95% CI)	*P*‐value	RR (95% CI)	*P*‐value	Risk (95% CI)	Proportion (95% CI)
No family history of psychosis	19 of 165 weeks (12%)	Reference		234 of 933 weeks (25%)	2.3 (1.3–4.3)	0.006	2.4 (1.3–4.2)	0.004				
Family history of psychosis	13 of 44 weeks (30%)	2.4 (0.9–7.0)	0.094	94 of 217 weeks (43%)	4.0 (2.0–7.9)	<0.001	1.5 (0.7–3.3)	0.267	0.7 (0.3–1.8)^c^	0.454	0.20 (−1.63–2.03)	0.05 (−0.42–0.52)

*Note*: All risk ratios have been adjusted for correlates of methamphetamine use: sex, days of cannabis use in the past week and days of tobacco use in the past week (see Table S2). Unadjusted estimates are comparable and can be found in Table S3. Abbreviation: RR, relative risk.

^a^ Relative excess risk due to the interaction.

^b^ Attributable Proportion: Proportion of psychosis cases among participants with both exposures (methamphetamine use and the modifier) that are due to the interaction.

^c^ In this model, the main effects for methamphetamine (RR = 2.3, 95% CI = 1.3–4.3) and family history of psychosis (RR = 2.4, 95% CI = 0.9–7.0) are the same as those presented in risk quadrants for each of these factors.

[Correction added on 14 June 2023, after first online publication: The number of weeks have been added under ‘No past week methamphetamine use’ and ‘Past week methamphetamine use’, for clarity in this version.]

**FIGURE 1:**
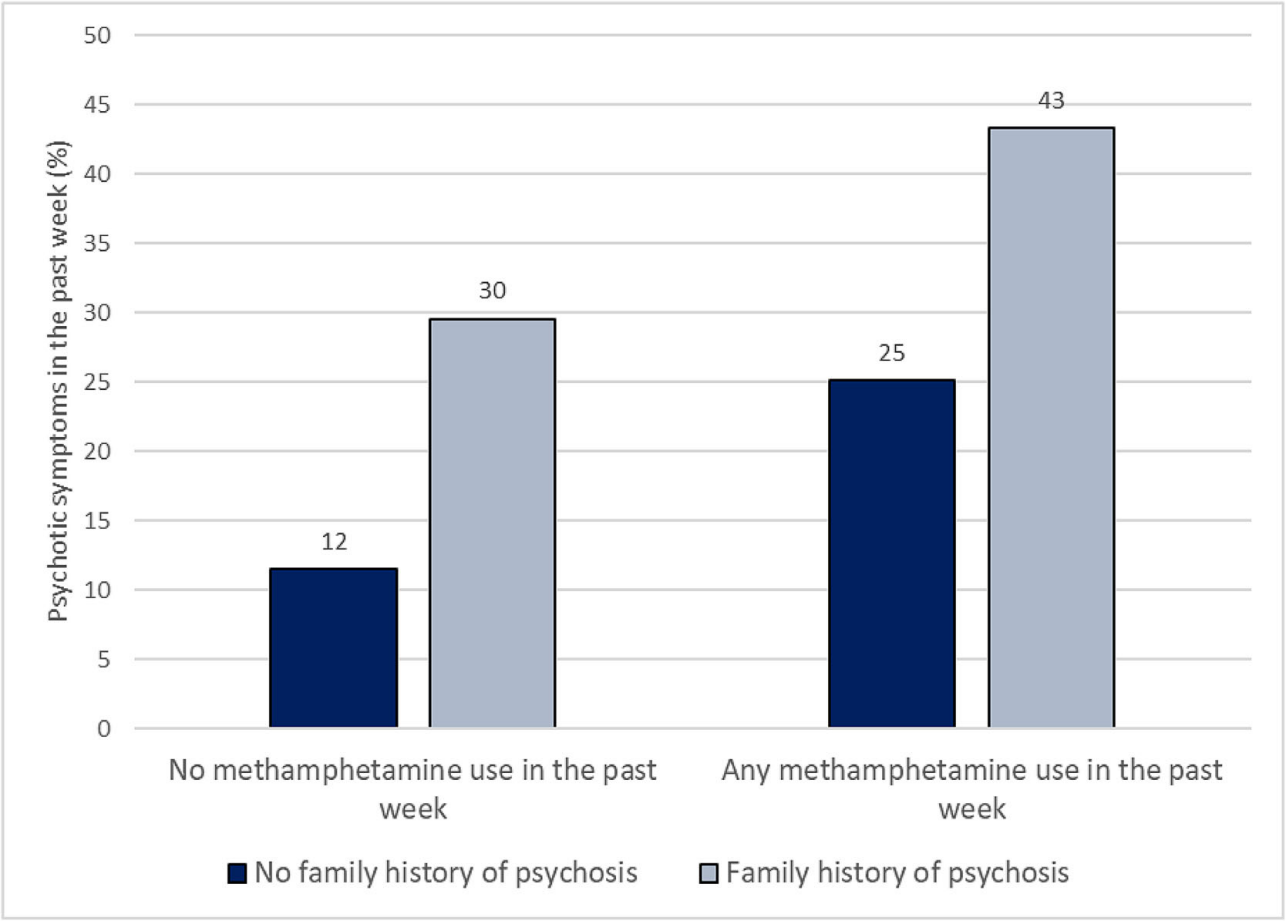
Relationship between past week methamphetamine use and past week psychotic symptoms by family history of psychosis.

The RR of psychotic symptoms for each quadrant in the effect modification analysis shows that methamphetamine and family history were independently associated with a risk of psychotic symptoms and that their joint risk was additive rather than multiplicative. Specifically, relative to participants with no family history of psychosis during weeks when they were not using methamphetamine (12% of which involved psychotic symptoms), the risk of psychotic symptoms was significantly greater during weeks when these participants were using methamphetamine (25% RR = 2.3, 95% CI = 1.3–4.3). For participants with a family history of psychosis, their risk of psychotic symptoms was significantly elevated relative to participants with no family history of psychosis even during weeks when they were not using methamphetamine (30%; RR = 2.4, 95% CI = 0.9–7.0). The highest risk of psychotic symptoms was seen for these participants during weeks when they were using methamphetamine (43%; RR = 4.0, 95% CI = 2.0–7.9) (Figure 1).

The non‐significant interaction (RR = 0.7, 95% CI = 0.3–1.8) indicates that the RR of psychotic symptoms during weeks of methamphetamine use did not differ significantly by whether people had a family history of psychosis. In fact, this interaction was slightly negative, which reflects that the RR of psychotic symptoms during weeks of methamphetamine use was slightly lower among participants with a family history of psychosis (RR = 1.5, 95% CI = 0.7–3.3) compared to participants with no family history of psychosis (RR = 2.4, 95% CI = 1.3–4.2).

Despite this non‐significant interaction effect, there was a small non‐significant positive excess risk (RERI = 0.20, 95% CI = −1.63 to 2.03), and an additional proportion of weeks involving psychotic symptoms (AP = 0.05, 95% CI = −0.42 to 0.52), for participants with a family history of psychosis. This seemingly contrary result is because of the higher baseline risk of psychotic symptoms among people with a family history of psychosis, which results in a higher absolute risk for a given risk ratio.

### Sensitivity analyses

Results of the sensitivity analyses can be found in the Supporting Information.

The analysis with imputed missing data (Tables S4–S6) found that cannabis was associated with psychotic symptoms (Table S4), but adjustment for cannabis use did not change the effect modification results (Table S6).

Substituting days of methamphetamine use in the past week as the predictor variable (rather than any past week methamphetamine use) produced comparable results in most respects (Tables S7–S10; Figure S2), except that the RERI (0.06, 95% CI = 0.03–0.12) and AP (0.03, 95% CI = 0.01–0.08) reached significance (Table S10). However, when missing data were imputed (Tables S11–S13), the RERI and AP were smaller and no longer significant (RERI = 0.03, 95% CI = –0.14 to 0.11; AP = 0.02, 95% CI = −0.05 to 0.08) (Table S14).

Removing weeks where participants took antipsychotic medication (removing 194 weeks of data from 19 participants) produced a similar pattern of results (Table S15), except that there was a significant negative interaction effect (RR = 0.4, 95% CI = 0.1–1.0, *P* = 0.042) reflecting a significantly larger RR of psychotic symptoms associated with methamphetamine use for participants who had no family history of psychosis (RR = 3.7, 95% 1.9–7.1; family history of psychosis RR = 1.3, 95% CI = 0.7–2.6). Similar results were found excluding participants who took any antipsychotic medication during the trial (Table S16).

Replacing the outcome variable with severity of psychotic symptoms in the past week did not change the results (Tables S17–S20).

## DISCUSSION

The evidence from our data is consistent with both methamphetamine use and family morbidity for psychosis being independent risk factors for psychotic symptoms among people dependent on methamphetamine (scenario 2). The joint risk associated with both factors resulted in a particularly high risk of psychotic symptoms among people who had a family history of psychosis during weeks when they were using methamphetamine (43% past week prevalence of psychotic symptoms). We did not find any evidence that having a family history of psychosis increased the RR of psychotic symptoms during weeks when methamphetamine was used (scenario 3). However, we found some limited evidence that a family history of psychosis may disproportionately contribute to cases of methamphetamine‐related psychotic symptoms (e.g. between 3% and 20% additional risk, accounting for up to 5% more weeks involving psychotic symptoms). Importantly, our data did not support scenario 1, in that the risk of psychotic symptoms during weeks of methamphetamine use was not confined to participants with a family history of psychosis.

These findings have important public health implications in showing that the risk of methamphetamine‐related psychotic symptoms is not confined to a minority of individuals with a family history of psychosis. This broadens the target group for interventions from people who are perceived to be at risk of psychosis to the broader group of methamphetamine consumers who may not ordinarily be engaged with mental health services or see themselves at risk of experiencing psychosis. It highlights the importance of assessing and responding to psychosis risk in people with methamphetamine use disorders within the alcohol and other drug sector, even if they do not have a personal or family history of schizophrenia.

Our findings run counter to the notion that methamphetamine use is solely exacerbating an underlying genetic vulnerability to psychosis. Not only was the risk of methamphetamine‐related psychotic symptoms not specific to people who had a family history of psychosis, but the risk of methamphetamine‐related psychotic symptoms was similar (if not larger) among people who had no family history of psychosis. Instead, our findings are more consistent with a drug‐induced psychosis brought about by heavy consumption of methamphetamine.

The joint additive risk posed by both methamphetamine and having a family history of psychosis resulted in a large risk of psychosis among people with a family history of psychosis during weeks when they were using methamphetamine. Importantly, these people remained at elevated risk of psychosis when they were not using methamphetamine. This could be because they experienced more prolonged psychotic symptoms in response to methamphetamine use (i.e. extending into periods of abstinence) [9, 10]. Alternatively, these symptoms could be psychotic experiences unrelated to drug use, reflecting a broader vulnerability to psychosis (e.g. undiagnosed or subthreshold schizophrenia). The more prevalent and persistent risk of psychotic symptoms seen among people with a family history of psychosis warrants more intensive support and continuing care, such as linkage with early intervention services, to reduce psychosis risk [39].

### Limitations

There were important risk factors for psychosis that were not assessed, including migration [40], urbanization [32, 41], ethnicity [40], older paternal age [42] and neonatal factors and childhood trauma [36]. Although we did not find any evidence that duration of methamphetamine use or early onset use affected psychosis vulnerability, for practical reasons, we were unable to assess the impact of historical use patterns in detail (e.g. lifetime exposure to heavy binge use), which may have produced a lasting vulnerability to psychosis.

Our findings are based on a sample of people dependent on methamphetamine who were using the drug daily or almost daily and who had no prior diagnosis of a primary psychotic disorder. They cannot necessarily be generalised to infrequent or recreational use of methamphetamine or people who have a diagnosed psychotic disorder. Participants were voluntarily taking part in a randomized controlled treatment trial and their characteristics may not generalize to the broader population of people who are dependent on methamphetamine.

Our analyses cannot identify the direction of the relationship between methamphetamine use and psychotic symptoms, although the broader evidence base points toward methamphetamine use contributing to the risk of psychosis [43]. Periods of abstinence from methamphetamine use were brief (past week) meaning that we were not able to distinguish between prolonged methamphetamine‐related psychotic symptoms and the psychotic experiences that were unrelated to the drug (e.g. an undiagnosed primary psychotic disorder). The threshold used to identify psychotic symptoms was also low (i.e. it included subclinical symptoms of psychosis). This may have led to a ceiling effect for participants at high risk of psychosis, and coupled with the limited sample size, may have been responsible for some of the non‐significant results seen for the risk of psychotic symptoms associated with a family history of psychosis.

Data on family history were based on self‐report, and although self‐report was obtained using a structured set of questions from the DIP [28], these reports were not verified by reports from family members or medical records. Our data were too sparse to examine the degree of familial morbidity for psychosis, or the specific contribution of bipolar disorder versus schizophrenia. We also included psychosis among first, second‐ or third‐degree relatives, resulting in a higher prevalence of familial morbidity compared to other studies that include only first‐degree relatives [9, 44]. This may have diluted associations between having a family history of psychosis and psychosis risk.

### CONCLUSIONS

Both methamphetamine use and having a family history of psychosis jointly contributed to the risk of psychotic symptoms in this sample of people dependent on methamphetamine. Having a family history of psychosis was not necessary for people to experience psychotic symptoms during weeks when they used methamphetamine. This broadens the pool of individuals at‐risk of methamphetamine‐related psychotic symptoms to include people who may not have any known family history of psychosis. The particularly high risk of psychotic symptoms seen among people with a family history of psychosis during weeks of methamphetamine use indicates a need for intensive support and continuing care to reduce their risk of psychosis.

## AUTHOR CONTRIBUTIONS

**Rebecca McKetin:** Conceptualization (equal); data curation (equal); formal analysis (equal); funding acquisition (lead); investigation (lead); methodology (equal); project administration (lead); writing—original draft (lead); writing—review and editing (lead). **Philip J. Clare:** Formal analysis (equal); methodology (supporting); writing—review and editing (supporting). **David Castle, Alyna Turner, Peter J. Kelly, Dan I. Lubman, Shalini Arunogiri, Victoria Manning, Michael Berk:** Conceptualization (supporting); writing—review and editing (supporting).

## DECLARATION OF INTERESTS

M.B. has received grant/research support from the National Institutes of Health, Cooperative Research Centre, Simons Autism Foundation, Cancer Council of Victoria, Stanley Medical Research Foundation, Medical Benefits Fund, National Health and Medical Research Council, Medical Research Futures Fund, Beyond Blue, Rotary Health, A2 milk company, Meat and Livestock Board, Woolworths, Avant and the Harry Windsor Foundation; has been a speaker for Abbot, Astra Zeneca, Janssen and Janssen, Lundbeck and Merck; and served as a consultant to Allergan, Astra Zeneca, Bioadvantex, Bionomics, Collaborative Medicinal Development, Eisai, Janssen and Janssen, Lundbeck Merck, Pfizer and Servier—all unrelated to this work. D.L. has provided consultancy advice to Lundbeck and Indivior and has received travel support and speaker honoraria from Astra Zeneca, Camurus, Indivior, Janssen, Lundbeck, Servier and Shire. S.A. has received speaker honoraria from Gilead, Janssen and Camurus for work unrelated to this study. The other authors have no interests to declare.

## Supporting information


**Table S1** Summary of missing data by variable
**Table S2** Correlates of any use of methamphetamine in the past week
**Table S3** Unadjusted effect modification analyses for whether a family history of psychosis modifies the risk of psychotic symptoms during weeks of methamphetamine use
**Table S4** Correlates of any psychotic symptom in the past week: imputed dataset
**Table S5** Correlates of any use of methamphetamine in the past week: imputed dataset
**Table S6** Effect modification analyses for whether a family history of psychosis modifies the risk of psychotic symptoms in the past week during weeks of methamphetamine use: imputed dataset
**Table S7** Correlates of days of methamphetamine use in the past week
**Table S8** Interaction effect for family history of psychosis and days of methamphetamine use in the past week in predicting psychotic symptoms in the past week
**Table S9** Risk of past week psychotic symptoms by days of methamphetamine use stratified by a family history of psychosis
**Table S10** RERI and AP for family history of psychosis and days of methamphetamine use in the past week in predicting psychotic symptoms in the past week
**Table S11** Correlates of days of methamphetamine use in the past week: imputed dataset
**Table S12** Interaction effect for family history of psychosis and days of methamphetamine use in the past week in predicting psychotic symptoms in the past week: imputed dataset
**Table S13** Risk of past week psychotic symptoms by days of methamphetamine use in the past week stratified by a family history of psychosis: imputed dataset
**Table S14** RERI and AP for family history of psychosis on days of methamphetamine use in the past week in predicting psychotic symptoms in the past week: imputed dataset
**Table S15** Sensitivity analysis of modification effects, excluding weeks where antipsychotic medication was taken (*n* = 138)
**Table S16** Sensitivity analysis of modification effects, excluding participants who took any antipsychotic mediation during the trial (*n* = 129)
**Table S17** Frequency and percentage of observations by most severe BPRS item rating
**Table S18** Correlates of the severity of psychotic symptoms experienced in the past week
**Table S19** The odds of more severe psychotic symptoms in the past week by days of methamphetamine use in the past week stratified by a family history of psychosis
**Table S20** Interaction effect for family history of psychosis and days of methamphetamine use in the past week in predicting the severity of psychotic symptoms in the past week
**Figure S1** Most common patterns of missing data
**Figure S2** Predicted probability of psychotic symptoms with days of methamphetamine use in the past week by a family history of psychosis

## Data Availability

Data are not publicly available. Please request access through the study team.
