# Supplementary material for: How does a family history of psychosis influence the risk of methamphetamine‐related psychotic symptoms: Evidence from longitudinal panel data
Source: Addiction. 2023 May 30;118(10):1975–83. doi: 10.1111/add.16230 (PMC10952942; doi:10.1111/add.16230)
Supplement: Supplementary file 1 — Table S1 Summary of missing data by variable Table S2 Correlates of any use of methamphetamine in the past week Table S3 Unadjusted effect modification analyses for whether a family history of psychosis modifies the risk of psychotic symptoms during weeks of methamphetamine use Table S4 Correlates of any psychotic symptom in the past week: imputed dataset Table S5 Correlates of any use of methamphetamine in the past week: imputed dataset Table S6 Effect modification analyses for whether a family history of psychosis modifies the risk of psychotic symptoms in the past week during weeks of methamphetamine use: imputed dataset Table S7 Correlates of days of methamphetamine use in the past week Table S8 Interaction effect for family history of psychosis and days of methamphetamine use in the past week in predicting psychotic symptoms in the past week Table S9 Risk of past week psychotic symptoms by days of methamphetamine use stratified by a family history of psychosis Table S10 RERI and AP for family history of psychosis and days of methamphetamine use in the past week in predicting psychotic symptoms in the past week Table S11 Correlates of days of methamphetamine use in the past week: imputed dataset Table S12 Interaction effect for family history of psychosis and days of methamphetamine use in the past week in predicting psychotic symptoms in the past week: imputed dataset Table S13 Risk of past week psychotic symptoms by days of methamphetamine use in the past week stratified by a family history of psychosis: imputed dataset Table S14 RERI and AP for family history of psychosis on days of methamphetamine use in the past week in predicting psychotic symptoms in the past week: imputed dataset Table S15 Sensitivity analysis of modification effects, excluding weeks where antipsychotic medication was taken (n = 138) Table S16 Sensitivity analysis of modification effects, excluding participants who took any antipsychotic mediation during the trial (n = 129) Table S17 Fre [file ADD-118-1975-s001.docx]

**Online appendix**

**Table of contents**

[1 List of Tables 2](#_Toc135239122)

[2 List of Figures 4](#_Toc135239123)

[3 Abbreviations 4](#_Toc135239124)

[4 STROBE Checklist 5](#_Toc135239125)

[5 Imputation methods 8](#_Toc135239126)

[6 Additional results tables for the main analysis 11](#_Toc135239127)

[7 Sensitivity analyses 13](#_Toc135239128)

[7.1 Sensitivity analysis using the imputed dataset 13](#_Toc135239129)

[7.2 Sensitivity analysis using days of methamphetamine use in the past week 16](#_Toc135239130)

[7.3 Sensitivity analysis for antipsychotic medication 21](#_Toc135239131)

[7.4 Sensitivity analysis using severity of psychotic symptoms 22](#_Toc135239132)

[8 References 25](#_Toc135239133)

# List of Tables

[Table S1 Summary of missing data by variable 9](#_Toc135239089)

[Table S2 Correlates of any use of methamphetamine in the past week 11](#_Toc135239090)

[Table S3 Unadjusted effect modification analyses for whether a family history of psychosis modifies the risk of psychotic symptoms during weeks of methamphetamine use 12](#_Toc135239091)

[Table S4 Correlates of any psychotic symptom in the past week: imputed dataset 13](#_Toc135239092)

[Table S5 Correlates of any use of methamphetamine in the past week: imputed dataset 14](#_Toc135239093)

[Table S6 Effect modification analyses for whether a family history of psychosis modifies the risk of psychotic symptoms in the past week during weeks of methamphetamine use: imputed dataset 15](#_Toc135239094)

[Table S7 Correlates of days of methamphetamine use in the past week 17](#_Toc135239095)

[Table S8 Interaction effect for family history of psychosis and days of methamphetamine use in the past week in predicting psychotic symptoms in the past week 17](#_Toc135239096)

[Table S9 Risk of past week psychotic symptoms by days of methamphetamine use stratified by a family history of psychosis 17](#_Toc135239097)

[Table S10 RERI and AP for family history of psychosis and days of methamphetamine use in the past week in predicting psychotic symptoms in the past week 18](#_Toc135239098)

[Table S11 Correlates of days of methamphetamine use in the past week: imputed dataset 19](#_Toc135239099)

[Table S12 Interaction effect for family history of psychosis and days of methamphetamine use in the past week in predicting psychotic symptoms in the past week: imputed dataset 19](#_Toc135239100)

[Table S13 Risk of past week psychotic symptoms by days of methamphetamine use in the past week stratified by a family history of psychosis: imputed dataset 19](#_Toc135239101)

[Table S14 RERI and AP for family history of psychosis on days of methamphetamine use in the past week in predicting psychotic symptoms in the past week: imputed dataset 20](#_Toc135239102)

[Table S15 Sensitivity analysis of modification effects, excluding weeks where antipsychotic medication was taken (n = 138) 21](#_Toc135239103)

[Table S16 Sensitivity analysis of modification effects, excluding participants who took any antipsychotic mediation during the trial (n = 129) 21](#_Toc135239104)

[Table S17 Frequency and percentage of observations by most severe BPRS item rating 23](#_Toc135239105)

[Table S18 Correlates of the severity of psychotic symptoms experienced in the past week 23](#_Toc135239106)

[Table S19 The odds of more severe psychotic symptoms in the past week by days of methamphetamine use in the past week stratified by a family history of psychosis 24](#_Toc135239107)

[Table S20 Interaction effect for family history of psychosis and days of methamphetamine use in the past week in predicting the severity of psychotic symptoms in the past week 24](#_Toc135239108)

# List of Figures

[Figure S1 Most common patterns of missing data 10](#_Toc128227483)

[Figure S2 Predicted probability of psychotic symptoms with days of methamphetamine use in the past week by a family history of psychosis 18](#_Toc128227484)

# Abbreviations

AP Attributable proportion of cases

CI Confidence interval

OR Odds ratio

RERI Relative excess risk due to the interaction

RR Risk ratio

# STROBE Checklist

|  | | **Item No** | **Recommendation** | **Page  No** |
| --- | --- | --- | --- | --- |
| **Title and abstract** | | 1 | (*a*) Indicate the study’s design with a commonly used term in the title or the abstract | 1&2 |
|  |  |  | (*b*) Provide in the abstract an informative and balanced summary of what was done and what was found | 2 |
| **Introduction** | | | | |
| Background/rationale | | 2 | Explain the scientific background and rationale for the investigation being reported | 3 |
| Objectives | | 3 | State specific objectives, including any prespecified hypotheses | 4 |
| **Methods** | | | | |
| Study design | | 4 | Present key elements of study design early in the paper | 5 |
| Setting | | 5 | Describe the setting, locations, and relevant dates, including periods of recruitment, exposure, follow-up, and data collection | 5 |
| Participants | | 6 | (*a*) *Cohort study*—Give the eligibility criteria, and the sources and methods of selection of participants. Describe methods of follow-up  *Case-control study*—Give the eligibility criteria, and the sources and methods of case ascertainment and control selection. Give the rationale for the choice of cases and controls  *Cross-sectional study*—Give the eligibility criteria, and the sources and methods of selection of participants | 5 |
|  |  |  | (*b*) *Cohort study*—For matched studies, give matching criteria and number of exposed and unexposed  *Case-control study*—For matched studies, give matching criteria and the number of controls per case | N/A |
| Variables | | 7 | Clearly define all outcomes, exposures, predictors, potential confounders, and effect modifiers. Give diagnostic criteria, if applicable | 6-7 |
| Data sources/ measurement | | 8* | For each variable of interest, give sources of data and details of methods of assessment (measurement). Describe comparability of assessment methods if there is more than one group | 6-7 |
| Bias | | 9 | Describe any efforts to address potential sources of bias | 9 |
| Study size | | 10 | Explain how the study size was arrived at | 5 |
| Quantitative variables | | 11 | Explain how quantitative variables were handled in the analyses. If applicable, describe which groupings were chosen and why | 7-9 |
| Statistical methods | | 12 | (*a*) Describe all statistical methods, including those used to control for confounding | 7-9 |
|  |  |  | (*b*) Describe any methods used to examine subgroups and interactions | 8-9 |
|  |  |  | (*c*) Explain how missing data were addressed | 9 |
|  |  |  | (*d*) *Cohort study*—If applicable, explain how loss to follow-up was addressed  *Case-control study*—If applicable, explain how matching of cases and controls was addressed  *Cross-sectional study*—If applicable, describe analytical methods taking account of sampling strategy | 9 |
|  |  |  | (*e*) Describe any sensitivity analyses | 9 |
| **Results** | | | | |
| Participants | 13* | (a) Report numbers of individuals at each stage of study—eg numbers potentially eligible, examined for eligibility, confirmed eligible, included in the study, completing follow-up, and analysed | | 6 |
|  |  | (b) Give reasons for non-participation at each stage | | N/A |
|  |  | (c) Consider use of a flow diagram | | N/A |
| Descriptive data | 14* | (a) Give characteristics of study participants (eg demographic, clinical, social) and information on exposures and potential confounders | | 10 |
|  |  | (b) Indicate number of participants with missing data for each variable of interest | | Table S1 |
|  |  | (c) *Cohort study*—Summarise follow-up time (eg, average and total amount) | | 6^a^ |
| Outcome data | 15* | *Cohort study*—Report numbers of outcome events or summary measures over time | | 10 & Table 1 |
|  |  | *Case-control study—*Report numbers in each exposure category, or summary measures of exposure | | N/A |
|  |  | *Cross-sectional study—*Report numbers of outcome events or summary measures | | N/A |
| Main results | 16 | (*a*) Give unadjusted estimates and, if applicable, confounder-adjusted estimates and their precision (eg, 95% confidence interval). Make clear which confounders were adjusted for and why they were included | | Table 2 |
|  |  | (*b*) Report category boundaries when continuous variables were categorized | | N/A |
|  |  | (*c*) If relevant, consider translating estimates of relative risk into absolute risk for a meaningful time period | | Table 2 |
| Other analyses | 17 | Report other analyses done—eg analyses of subgroups and interactions, and sensitivity analyses | | 12 |
| **Discussion** | | | | |
| Key results | 18 | Summarise key results with reference to study objectives | | 13 |
| Limitations | 19 | Discuss limitations of the study, taking into account sources of potential bias or imprecision. Discuss both direction and magnitude of any potential bias | | 14-15 |
| Interpretation | 20 | Give a cautious overall interpretation of results considering objectives, limitations, multiplicity of analyses, results from similar studies, and other relevant evidence | | 13-15 |
| Generalisability | 21 | Discuss the generalisability (external validity) of the study results | | 15 |
| **Other information** | | | | |
| Funding | 22 | Give the source of funding and the role of the funders for the present study and, if applicable, for the original study on which the present article is based | | 16 |

^a^Readers are also referred to other papers which provide this information in detail.

*Give information separately for cases and controls in case-control studies and, if applicable, for exposed and unexposed groups in cohort and cross-sectional studies.

**Note:** An Explanation and Elaboration article discusses each checklist item and gives methodological background and published examples of transparent reporting. The STROBE checklist is best used in conjunction with this article (freely available on the Web sites of PLoS Medicine at http://www.plosmedicine.org/, Annals of Internal Medicine at http://www.annals.org/, and Epidemiology at http://www.epidem.com/). Information on the STROBE Initiative is available at www.strobe-statement.org.

# Imputation methods

The data contained a relatively large number of missing data points, both because of attrition/intermittent completion of waves, and also because not all participants answered every question in each survey. Information about the amount and most common patterns of missing data is included in Table S1 and Figure S1.

Data was confirmed to be not missing completely at random via Little’s test. As such, we have assumed the data to be missing at random. Because missingness can introduce bias when there is missingness in both the outcome and exposure variables^1^ that is not completely at random^2^, we conducted a sensitivity analysis using multiple imputation.

We conducted the imputation using fully conditional specification (FCS; also called chained equations) in R (ver. 4.0.3).^3^ Consistent with recommendations in the literature, we first attempted to impute using the ‘just another variable’ approach,^4^ treating repeated measurements as individual variables. However, this approach failed to converge. As such, we used traditional FCS, which has been shown to perform as well in longitudinal data as more complex methods.^4,5^ To allow for possible non-linear effects and interactions in the imputation, we imputed using random forests.^6^

Consistent with the principle of compatibility between the imputation and outcome models, which states that multiple imputation can introduce bias if the imputation model does not include variables or other information that is used in the outcome models,^2^ imputation models contained all variables used in the outcome models, as well as the clustering (id) variable. Analyses were then conducted on each imputed dataset and combined using Rubin’s rules. We used M=20 imputations^7^.

Table S1 Summary of missing data by variable

| Variable | Variable description | Missing data in variable | |
| --- | --- | --- | --- |
|  |  | n | % |
| depwk | Antidepressant use in the past week (days) | 561 | 29.1 |
| alcwk | Alcohol use in the past week (days) | 560 | 29.0 |
| benwk | Bezodiazepine use in the past week(days) | 560 | 29.0 |
| herwk | Heroin use in the past week(days) | 559 | 29.0 |
| opiwk | Other opioid use in the past week (days) | 559 | 29.0 |
| cocwk | Cocaine use in the past week (days) | 559 | 29.0 |
| ecswk | Ecstasy use in the past week (days) | 559 | 29.0 |
| halwk | Hallucinogen use in the past week (days) | 559 | 29.0 |
| inhwk | Inhalant use in the past week (days) | 559 | 29.0 |
| canwk | Cannabis use in the past week (days) | 559 | 29.0 |
| tobwk | Tobacco use in the past week (days) | 559 | 29.0 |
| antwk | Antipsychotic use in the past week (days) | 559 | 29.0 |
| susscore | BPRS Suspiciousness score | 558 | 28.9 |
| delscore | BPRS unusual thought content score | 558 | 28.9 |
| halscore | BPRS hallucinations score | 558 | 28.9 |
| methwk | Days of methamphetamine use in the past week | 558 | 28.9 |
| family_schiz | Family history of schizophrenia | 13 | 0.7 |
| family_bipolar | Family history of bipolar | 13 | 0.7 |
| base_awq | Amphetamine Withdrawal Questionnaire score | 13 | 0.7 |


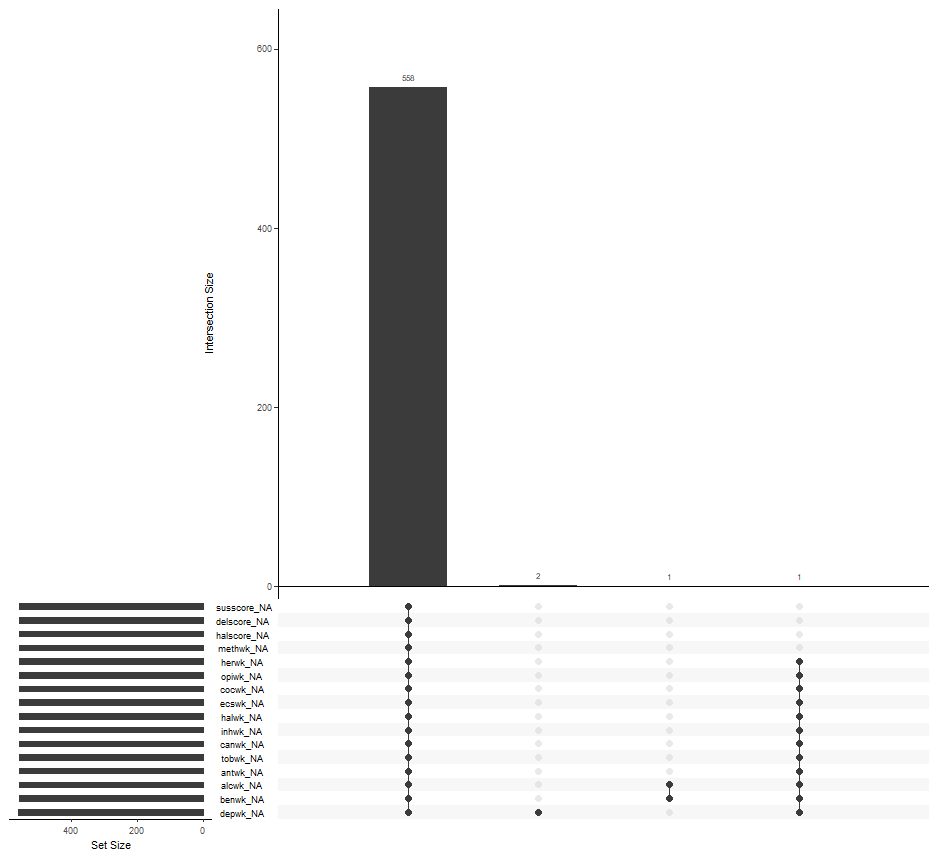


Figure S1 Most common patterns of missing data

# Additional results tables for the main analysis

Table S2 Correlates of any use of methamphetamine in the past week

|  | Any methamphetamine use in the past week^a^ |  |
| --- | --- | --- |
|  | RR (95% CI) | P value |
| **Time-invariant baseline variables** |  |  |
| Family history of psychosis | 0.98 (0.86 – 1.11) | 0.739 |
| Male | 0.89 (0.81 – 0.97) | 0.008 |
| Age | 1.00 (1.00 -1.01) | 0.096 |
| Years of schooling | 0.98 (0.96 – 1.01) | 0.201 |
| Unemployed | 1.02 (0.93 – 1.12) | 0.683 |
| Income^a^ |  |  |
| < 400 | Reference |  |
| 400-799 | 1.17 (0.95 – 1.43) | 0.144 |
| 800-1199 | 1.08 (0.85 – 1.37) | 0.524 |
| 1200+ | 1.06 (0.84 – 1.34) | 0.648 |
| Age of first methamphetamine use (years) | 1.00 (1.00 – 1.01) | 0.117 |
| Duration of methamphetamine use (years) | 1.00 (1.00 -1.01) | 0.623 |
| Injecting methamphetamine use | 0.94 (0.84 – 1.04) | 0.229 |
| **Time-varying drug use in the past week^b^** |  |  |
| Any cannabis use | 1.18 (1.05 – 1.33) | 0.006 |
| Days of cannabis use | 1.03 (1.01 – 1.05) | 0.002 |
| Any alcohol use | 1.00 (0.95 – 1.04) | 0.840 |
| Days of alcohol use | 1.00 (0.98 – 1.01) | 0.688 |
| Any tobacco use | 1.02 (0.90 – 1.16) | 0.752 |
| Days of tobacco use | 1.02 (1.00 – 1.03) | 0.016 |
| Any other drug use^c^ | 1.05 (0.98 -1.12) | 0.160 |

^b^AU$ net per fortnight

^a^ Based on time-varying data collected at each weekly assessment for the past week

^c^Use of any other drug class (cocaine, ecstasy, hallucinogens, inhalants heroin or other opioids)

Table S3 Unadjusted effect modification analyses for whether a family history of psychosis modifies the risk of psychotic symptoms during weeks of methamphetamine use

|  | No past week methamphetamine use | | | Past week methamphetamine use | | | Within strata effect |  | Interaction effect ^a^ |  | Relative excess risk due to the interaction (RERI)^b^ | Attributable proportion of cases (AP)^c^ |
| --- | --- | --- | --- | --- | --- | --- | --- | --- | --- | --- | --- | --- |
|  | Past week psychotic symptoms, n (%) | RR (95% CI) | P value | Past week psychotic symptoms, n (%) | RR (95% CI) | P value | RR (95% CI) | P value | RR (95% CI) | P value | Risk (95% CI) | Proportion (95% CI) |
| No family history of psychosis | 19 (12) | Reference |  | 234 (25) | 2.3 (1.3 - 4.3) | 0.005 | 2.3 (1.3 - 4.3) | 0.005 |  |  |  |  |
| Family history of psychosis | 13 (30) | 2.4 (0.9 - 7.0) | 0.095 | 94 (43) | 4.0 (2.0 -7.9) | <0.001 | 1.6 (0.8 – 3.4) | 0.208 | 0.7 (0.3 – 1.8) | 0.446 | 0.19 (-1.61 – 1.98) | 0.05 (-0.41 - 0.51) |

^a^In this model, the main effects for methamphetamine and family history of psychosis are the same as those presented in risk quadrants for each of these factors.

^b^Relative excess risk due to the interaction.

^c^Attributable Proportion: Proportion of psychosis cases among participants with both exposures (methamphetamine use and the modifier) that are due to the interaction

# Sensitivity analyses

## Sensitivity analysis using the imputed dataset

Using the imputed dataset, psychotic symptoms were additionally associated with past week cannabis use (both any use and days of use in the past week) and other drug use (Table S4). All subsequent adjusted analyses adjusted for these variables as well as variables correlated with past week methamphetamine use (Table S5). Findings from the effect modification analyses (Table S6) were comparable to those from the unimputed dataset.

Table S4 Correlates of any psychotic symptom in the past week: imputed dataset

|  | Any psychotic symptoms in the past week |  |
| --- | --- | --- |
|  | RR (95% CI) | P value |
| **Time invariant (baseline) variables** |  |  |
| Family history of psychosis | 1.69 (1.20 - 2.38) | 0.003 |
| Male | 1.04 (0.75 - 1.44) | 0.818 |
| Age | 0.99 (0.97 - 1.01) | 0.241 |
| Years of schooling^a^ | 1.07 (0.96 - 1.19) | 0.235 |
| Unemployed | 1.03 (0.74 - 1.44) | 0.852 |
| Income^b^ |  |  |
| < 400 | Reference |  |
| 400-799 | 1.05 (0.66 - 1.67) | 0.839 |
| 800-1199 | 0.80 (0.45 - 1.45) | 0.471 |
| 1200+ | 0.95 (0.54 - 1.67) | 0.854 |
| Age of first methamphetamine use | 1.00 (0.98 - 1.02) | 0.751 |
| Duration of use | 0.99 (0.98 - 1.01) | 0.365 |
| **Past week drug use (time-varying)^c^** |  |  |
| Any methamphetamine use | 2.01 (1.25 - 3.23) | 0.004 |
| Days of methamphetamine use | 1.10 (1.04 - 1.16) | 0.001 |
| Any cannabis use | 1.50 (1.12 - 2.00) | 0.007 |
| Days of cannabis use | 1.06 (1.01 - 1.12) | 0.020 |
| Any alcohol use | 1.14 (0.93 - 1.41) | 0.206 |
| Days of alcohol use | 1.02 (0.97 - 1.08) | 0.468 |
| Any tobacco use | 1.14 (0.93 - 1.41) | 0.950 |
| Days of tobacco use | 0.99 (0.95 - 1.04) | 0.783 |
| Any other drug use^d^ | 1.46 (1.11 - 1.93) | 0.007 |

^a^AU$ net per fortnight

^b^12 years of schooling reflects completion of high school

^c^ Based on time-varying data for each week

^d^Use of any other drug class (cocaine, ecstasy, hallucinogens, inhalants heroin or other opioids)

Table S5 Correlates of any use of methamphetamine in the past week: imputed dataset

|  | Any use of methamphetamine in the past week |  |
| --- | --- | --- |
|  | RR (95% CI) | P value |
| **Time invariant (baseline) variables** |  |  |
| Family history of psychosis | 0.97 (0.86 – 1.11) | 0.688 |
| Male | 0.90 (0.83 – 0.98) | 0.015 |
| Age | 1.01 (1.00 – 1.01) | 0.043 |
| Years of schooling | 0.98 (0.96 – 1.01) | 0.239 |
| Unemployed | 1.02 (0.93 – 1.12) | 0.738 |
| Income^b^ |  |  |
| < 400 | Reference |  |
| 400-799 | 1.17 (0.96 – 1.41) | 0.119 |
| 800-1199 | 1.09 (0.87 – 1.36) | 0.436 |
| 1200+ | 1.06 (0.85 – 1.33) | 0.594 |
| Age of first methamphetamine use (years) | 1.00 (1.00 – 1.01) | 0.096 |
| Duration of use (years) | 1.00 (1.00 – 1.01) | 0.480 |
| **Past week drug use (time varying)^c^** |  |  |
| Any cannabis use | 1.13 (1.04 – 1.22) | 0.003 |
| Days of cannabis use | 1.02 (1.01 – 1.04) | 0.001 |
| Any alcohol use | 0.99 (0.94 – 1.06) | 0.857 |
| Days of alcohol use | 1.00 (0.98 – 1.02) | 0.744 |
| Any tobacco use | 1.07 (0.96 – 1.20) | 0.242 |
| Days of tobacco use | 1.02 (1.00 – 1.03) | 0.054 |
| Any other drug use^d^ | 1.05 (0.98 – 1.12) | 0.151 |

^a^AU$ net per fortnight

^b^12 years of schooling reflects completion of high school
^c^ Based on time-varying data for each week

^d^Use of any other drug class (cocaine, ecstasy, hallucinogens, inhalants heroin or other opioids)

Table S6 Effect modification analyses for whether a family history of psychosis modifies the risk of psychotic symptoms in the past week during weeks of methamphetamine use: imputed dataset

| **Unadjusted analysis** | | | | | | | | | | |
| --- | --- | --- | --- | --- | --- | --- | --- | --- | --- | --- |
|  | Risk of psychotic symptoms – unadjusted analysis | | | |  |  |  |  |  |  |
|  | No past week methamphetamine use | | Past week methamphetamine use | | Within strata effect |  | Interaction effect |  | Relative excess risk (RERI)^a^ | Attributable proportion of cases (AP)^b^ |
|  | RR (95% CI) | P value | RR (95% CI) | P value | RR (95% CI) | P value | RR (95% CI) | P value | Risk (95% CI) | Proportion (95% CI) |
| No family history of psychosis | Reference |  | 2.2 (1.3 - 3.9) | 0.005 | 2.2 (1.3 -4.0) | 0.005 |  |  |  |  |
| Family history of psychosis | 2.3 (0.8 - 6.6) | 0.119 | 3.7 (1.9 - 7.2) | < .001 | 1.6 (0.7 - 3.6) | 0.235 | 0.7 (0.3 - 1.9) | 0.509 | 0.17 (-2.22 – 1.92) | 0.04 (-0.45 – 0.49) |
| **Adjusted analysis^** | | | | | | | | | | |
|  | Risk of psychotic symptoms | | | |  |  |  |  |  |  |
|  | No past week methamphetamine use | | Past week methamphetamine use | | Within strata effect |  | Interaction effect |  | Relative excess risk (RERI)^a^ | Attributable proportion of cases (AP)^b^ |
|  | RR (95% CI) | P value | RR (95% CI) | P value | RR (95% CI) | P value | RR (95% CI) | P value | Risk (95% CI) | Proportion (95% CI) |
| No family history of psychosis | Reference |  | 2.2 (1.2 - 3.8) | 0.006 | 2.2 (1.3 -3.8) | 0.005 |  |  |  |  |
| Family history of psychosis | 2.1 (0.8 - 5.8) | 0.129 | 3.4 (1.8 - 6.4) | <0.001 | 1.5 (0.8 – 2.9) | 0.203 | 0.7 (0.3 - 1.9) | 0.516 | 0.10 (-2.05 –1.75) | 0.03 (-0.43 – 0.49) |

^Adjusted for sex, days of cannabis use in the past week, days of tobacco use in the past week, and other drug use (i.e., the use of any other drug classes in the past week, yes/no).

## Sensitivity analysis using days of methamphetamine use in the past week

Analyses were replicated replacing the predictor variable of any vs. no use of methamphetamine in the past week with a continuous variable of days of methamphetamine use in the past week. RERIs and APs were calculated using the methods proposed by Knol et al.^8^ and confidence limits were bootstrapped using non-parametric percentile bootstrapping methods.

Correlates of days of methamphetamine use in the past week included more days of cannabis use and there was a non-significant trend toward fewer years of schooling (Table S7). Subsequent adjusted analyses were adjusted for these variables and also variables associated with psychotic symptoms (Table 1 in the main paper). There was no significant interaction between having a family history of psychosis and days of methamphetamine use in the past week in predicting psychotic symptoms in the past week in either the unadjusted or adjusted analyses (Table S8), reflecting that the relative increase in the risk of psychotic symptoms with more days of methamphetamine use was not significantly different for participants with and without a family history of psychosis (Table S9). However, the higher baseline rate of psychotic symptoms amongst people with a family history of psychosis meant that there was an additive contribution to the absolute risk of psychosis amongst people with a family history of psychosis, with more days of methamphetamine use contributing to more risk and more cases of psychotic symptoms (i.e., positive RERI and AP; Table S10). For every day of methamphetamine use, participants with a family history of psychosis would experience an excess risk of 6%, accounting for an additional 3% of weeks involving psychotic symptoms. This relationship is depicted in Figure S2.

These analyses were re-run on the imputed dataset. Adjusted analyses included both variables correlated with psychotic symptoms in the imputed dataset (Table S4) and correlates of days of methamphetamine use in the past week in the imputed dataset (Table S11). Although the results (Tables S12 and S13) were similar to the unimputed analysis in most respects, the RERI and AP were smaller and not statistically significant in this analysis (Table S14).

Table S7 Correlates of days of methamphetamine use in the past week

|  | Days of methamphetamine use in the past week |  |
| --- | --- | --- |
|  | RR (95% CI) | P value |
| **Time invariant (baseline) variables** |  |  |
| Family history of psychosis | 1.03 (0.85 – 1.26) | 0.760 |
| Male | 0.89 (0.76 -1.03) | 0.121 |
| Age | 1.00 (0.99 -1.01) | 0.920 |
| Years of schooling | 0.96 (0.92 -1.00) | 0.051 |
| Unemployed | 0.95 (0.81 -1.11) | 0.507 |
| Income^b^ |  |  |
| < 400 | Reference |  |
| 400-799 | 1.09 (0.81 – 1.47) | 0.560 |
| 800-1199 | 1.09 (0.78 – 1.53) | 0.610 |
| 1200+ | 1.17 (0.84 – 1.61) | 0.351 |
| Age of first methamphetamine use (years) | 1.00 (0.99 -1.01) | 0.938 |
| Duration of use (years) | 1.00 (0.99 -1.01) | 0.973 |
| **Past week drug use (time varying)^a^** |  |  |
| Any cannabis use | 1.11 (0.97 – 1.28) | 0.133 |
| Days of cannabis use | 1.05 (1.02 -1.08) | < 0.001 |
| Any alcohol use | 0.97 (0.91 – 1.03) | 0.270 |
| Days of alcohol use | 1.01 (0.99 -1.04) | 0.331 |
| Any tobacco use | 0.95 (0.78 – 1.16) | 0.613 |
| Days of tobacco use | 1.02 (1.00 -1.04) | 0.056 |
| Any other drug use^d^ | 1.07 (0.98 – 1.17) | 0.153 |

^a^ Based on time-varying data for each week

^b^AU$ net per fortnight

^c^12 years of schooling reflects completion of high school

^d^Use of any other drug class (cocaine, ecstasy, hallucinogens, inhalants heroin or other opioids)

Table S8 Interaction effect for family history of psychosis and days of methamphetamine use in the past week in predicting psychotic symptoms in the past week

|  | Days of methamphetamine use in the past week | | Family history of psychosis | | Interaction | |
| --- | --- | --- | --- | --- | --- | --- |
|  | RR (95% CI) | P value | RR (95% CI) | P value | RR (95% CI) | P value |
| Unadjusted analysis | 1.11 (1.04 – 1.18) | < 0.001 | 1.81 (0.88 – 3.73) | 0.107 | 0.99 (0.89 – 1.10) | 0.822 |
| Adjusted analysis^ | 1.11 (1.05 – 1.18) | < 0.001 | 1.80 (0.88 – 3.68) | 0.105 | 0.98 (0.89 – 1.09) | 0.742 |

^Adjusted sex, days of cannabis use in the past week, days of tobacco use in the past week, and years of schooling

Table S9 Risk of past week psychotic symptoms by days of methamphetamine use stratified by a family history of psychosis

|  | Relative Risk (RR) of past week psychotic symptoms | |
| --- | --- | --- |
|  | RR (95% CI) | P value |
| Unadjusted analysis |  |  |
| No family history of psychosis | 1.11 (1.05 - 1.18) | < 0.001 |
| Family history of psychosis | 1.10 (1.01 - 1.20) | 0.037 |
| Adjusted analysis^ |  |  |
| No family history of psychosis | 1.12 (1.05 - 1.18) | < 0.001 |
| Family history of psychosis | 1.08 (0.99 - 1.18) | 0.098 |

^Adjusted sex, days of cannabis use in the past week, days of tobacco use in the past week and years of

Table S10 RERI and AP for family history of psychosis and days of methamphetamine use in the past week in predicting psychotic symptoms in the past week

|  | RERI (95% CI) | AP (95% CI) |
| --- | --- | --- |
| Unadjusted analysis | 0.07 (0.02 - 0.12) | 0.03 (0.02 - 0.09) |
| Adjusted analysis^ | 0.06 (0.03 – 0.12) | 0.03 (0.01 – 0.08) |

^Adjusted sex, days of cannabis use in the past week, days of tobacco use in the past week and years of schooling


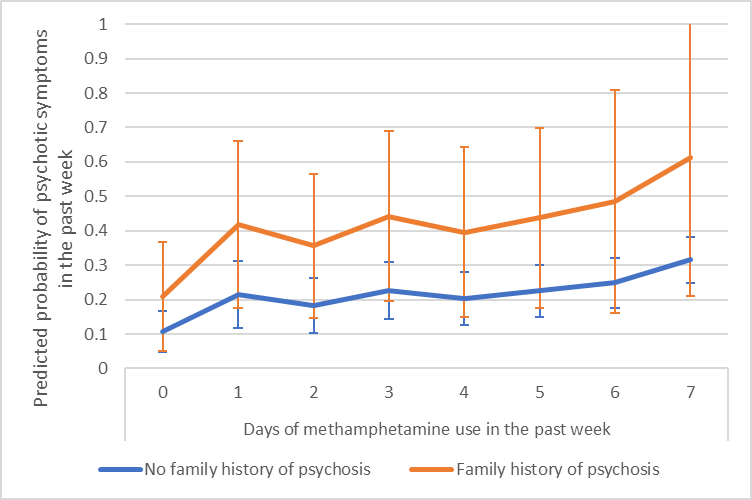


**Figure S2 Predicted probability of psychotic symptoms with days of methamphetamine use in the past week by a family history of psychosis**

Note. Margins extracted from adjusted model in Table S4. 95% confidence limits were derived using the delta method.

Table S11 Correlates of days of methamphetamine use in the past week: imputed dataset

|  | Days of methamphetamine use in the past week | |
| --- | --- | --- |
|  | RR (95% CI) | P value |
| **Time invariant (baseline) variables** |  |  |
| Family history of psychosis | 1.00 (0.82 – 1.21) | 0.978 |
| Male | 0.89 (0.77 –1.02) | 0.104 |
| Age | 1.00 (0.99 – 1.01) | 0.702 |
| Years of schooling | 0.96 (0.92 –1.00) | 0.051 |
| Unemployed | 0.94 (0.82 –1.09) | 0.433 |
| Income^b^ |  |  |
| < 400 | Reference |  |
| 400-799 | 1.09 (0.84 – 1.43) | 0.510 |
| 800-1199 | 1.12 (0.82 – 1.53) | 0.462 |
| 1200+ | 1.17 (0.87 –1.58) | 0.292 |
| Age of first methamphetamine use (years) | 1.00 (0.99 –1.01) | 0.845 |
| Duration of use (years) | 1.00 (0.99 – 1.01) | 0.824 |
| **Past week drug use (time varying)^a^** |  |  |
| Any cannabis use | 1.06 (0.95 – 1.19) | 0.269 |
| Days of cannabis use | 1.03 (1.01 – 1.05) | 0.006 |
| Any alcohol use | 0.96 (0.89 – 1.03) | 0.276 |
| Days of alcohol use | 1.00 (0.98 – 1.03) | 0.826 |
| Any tobacco use | 0.99 (0.84 – 1.16) | 0.876 |
| Days of tobacco use | 1.01 (0.99 – 1.03) | 0.333 |
| Any other drug use^d^ | 1.05 (0.95 – 1.15) | 0.349 |

Table S12 Interaction effect for family history of psychosis and days of methamphetamine use in the past week in predicting psychotic symptoms in the past week: imputed dataset

|  | Days of methamphetamine use in the past week | | Family history of psychosis | | Interaction | |
| --- | --- | --- | --- | --- | --- | --- |
|  | RR (95% CI) | P value | RR (95% CI) | P value | RR (95% CI) | P value |
| Unadjusted analysis | 1.10 (1.03 – 1.17) | 0.004 | 1.79 (0.87 – 3.67) | 0.113 | 0.99 (0.88 – 1.11) | 0.831 |
| Adjusted analysis^ | 1.09 (1.03 – 1.17) | 0.006 | 1.74 (0.86 – 3.51) | 0.122 | 0.98 (0.88 – 1.10) | 0.724 |

^Adjusted days of cannabis use in the past week, other drug use in the past week and years of schooling

Table S13 Risk of past week psychotic symptoms by days of methamphetamine use in the past week stratified by a family history of psychosis: imputed dataset

|  | Relative Risk (RR) of past week psychotic symptoms | |
| --- | --- | --- |
|  | RR (95% CI) | P value |
| Unadjusted analysis: |  |  |
| No family history of psychosis | 1.10 (1.03 – 1.17) | 0.004 |
| Family history of psychosis | 1.09 (0.98 – 1.20) | 0.103 |
| Adjusted analysis:^ |  |  |
| No family history of psychosis | 1.10 (1.03 – 1.17) | 0.005 |
| Family history of psychosis | 1.06 (0.96 – 1.18) | 0.241 |

^Adjusted sex, days of cannabis use in the past week, days of tobacco use in the past week and years of schooling

Table S14 RERI and AP for family history of psychosis on days of methamphetamine use in the past week in predicting psychotic symptoms in the past week: imputed dataset

|  | RERI (95% CI) | AP (95% CI) |
| --- | --- | --- |
| Unadjusted analysis | 0.05 (-0.12 – 0.14) | 0.03 (-0.04 – 0.09) |
| Adjusted analysis^ | 0.03 (-0.14 – 0.11) | 0.02 (-0.05 – 0.08) |

^Adjusted days of cannabis use in the past week, other drug use, and years of schooling

## Sensitivity analysis for antipsychotic medication

Sensitivity analyses excluding participants who took antipsychotic medication are shown in Tables S15 and S16. Nineteen participants took medication during the trial, affecting 194 (10%) of all weekly assessments: 5 of 27 with a family history of psychosis (19%) and 14 of 108 (11%) of participants without a family history of psychosis (Chi square = 0.99, p = 0.321). Participants with a family history of psychosis took antipsychotics during 54 or 350 weekly assessments (15%) compared to 140 of 1578 (9%) of weekly assessments for participants with no family history of psychosis (RR 1.7, 95% CI 0.6 - 4.6, p = 0.276). Excluding weeks where participants took antipsychotic medication resulted in excluding all data from 11 participants (3 with a family history of psychosis).

Table S15 Sensitivity analysis of modification effects, excluding weeks where antipsychotic medication was taken (n = 138)

|  | No methamphetamine use in the past week | | | Methamphetamine use in the past week | | | Within strata effect |  | Interaction effect |  | Relative excess risk (RERI)^a^ | Attributable proportion of cases (AP)^b^ |
| --- | --- | --- | --- | --- | --- | --- | --- | --- | --- | --- | --- | --- |
|  | Psychotic symptoms in the past week, n (%) | RR (95% CI) | P value | Psychotic symptoms in the past week, n (%) | RR (95% CI) | P value | RR (95% CI) | P value | RR (95% CI) | P value | Risk (95% CI) | Proportion (95% CI) |
| No family history of psychosis | 10 (7) | Reference |  | 205 (25) | 3.6 (1.8 - 7.1) | < 0.001 | 3.7 (1.9 - 7.1) | < 0.001 | Reference |  |  |  |
| Family history of psychosis | 13 (34) | 4.5 (1.6 - 12.6) | 0.004 | 81 (45) | 6.1 (2.9 - 12.9) | < 0.001 | 1.3 (0.7 - 2.6) | 0.405 | 0.4 (0.1 - 1.0) | 0.042 | -1.03 (-4.33 - 2.26) | -0.17 (-0.68 - 0.34) |

Note. Based on 1,214 weeks (observations). All rate ratios are adjusted for sex, days of cannabis use in the past week, and days of tobacco use in the past week.

Table S16 Sensitivity analysis of modification effects, excluding participants who took any antipsychotic mediation during the trial (n = 129)

|  | No methamphetamine use in the past week | | | Methamphetamine use in the past week | | | Within strata effect |  | Interaction effect |  | Relative excess risk (RERI)^a^ | Attributable proportion of cases (AP)^b^ |
| --- | --- | --- | --- | --- | --- | --- | --- | --- | --- | --- | --- | --- |
|  |  |  |  |  |  |  |  |  |  |  |  |  |
|  | Psychotic symptoms in the past week, n (%) | RR (95% CI) | P value | Psychotic symptoms in the past week, n (%) | RR (95% CI) | P value | RR (95% CI) | P value | RR (95% CI) | P value | Risk (95% CI) | Proportion (95% CI) |
| No family history of psychosis | 9 (6) | Reference |  | 200 (25) | 4.4 (2.0 – 9.7) | < 0.001 | 4.4 (2.0 – 9.6) | < 0.001 | Reference |  |  |  |
| Family history of psychosis | 13 (39) | 6.2 (2.1 – 18..4) | 0.001 | 81 (46) | 7.9 (3.4 – 18.5) | < 0.001 | 1.2 (0.6 - 2.4) | 0.522 | 0.3 (0.1 – 0.8) | 0.019 | -1.68 (-6.22 – 2.86) | -0.21 (-0.74 - 0.31) |

Note. Based on 1,214 weeks (observations). All rate ratios are adjusted for sex, days of cannabis use in the past week, and days of tobacco use in the past week.

## Sensitivity analysis using severity of psychotic symptoms

This sensitivity analysis involved using severity of psychotic symptoms rated on the BPRS rather than using a cut-off of 3 or more to identify participants with psychotic symptoms. Symptoms on the BPRS are rated against anchor points on a scale of 1 to 7 reflecting no symptoms (1) through to extremely severe symptoms (7). For this analysis, severity was graded according to the most severe symptom that a participant had during the past week on any of the three BPRS items of hallucinations, delusions and suspiciousness. The frequency of observations for each severity category on the BPRS is provided in Table S17. For the analysis, ratings of severe (BPRS score of 6) and extremely severe (BPRS score of 7) were combined because only three observations involved extremely severe symptoms. Scores were recalibrated to 0-5 by subtracting one.

To examine correlates of symptom severity we used an ordinal logistic regression model (with a random intercept to account for clustering of repeated measures over time and robust error estimates). An ordinal regression was used because the anchor points for BPRS ratings represent a ranking of symptom severity rather than a continuous score or discrete event. The outcome variable in each model was time-varying severity of psychotic symptoms in the past week. As can be seen in Table S18, the severity of psychotic symptoms in the past week was significantly correlated with past week methamphetamine use (both any past week use and days of use in the past week) and having a family history of psychosis. There was a significant correlation with other drug use in the past week and trends toward trends toward younger age and past week cannabis use being associated with the severity of psychotic symptoms in the past week. Subsequent adjusted analyses were adjusted for other drug use, any cannabis use in the past week and age, as well as correlates of methamphetamine use (i.e., years of schooling, days of cannabis use in the past week and days of tobacco use in the past week).

The magnitude of the relationship between days of methamphetamine use in the past week and severity of psychotic symptoms in the past week was the same for both participants with and without a family history of psychosis (Table S19). Neither the unadjusted nor the adjusted models showed any evidence of an interaction between days of methamphetamine use in the past week and a family history of psychosis in predicting the severity of psychotic symptoms in the past week (Table S20). The adjusted RERI for this interaction was 0.35 (95% CI -0.12 - 0.52) and the adjusted AP was 0.11 (95% CI -0.33 - 0.12): neither were statistically significant.

Table S17 Frequency and percentage of observations by most severe BPRS item rating

| **BPRS descriptor** | **BPRS item score** | **n** | **%** |
| --- | --- | --- | --- |
| Nil | 1 | 766 | 56 |
| Very mild | 2 | 233 | 17 |
| Mild | 3 | 227 | 17 |
| Moderate | 4 | 71 | 5 |
| Moderately severe | 5 | 47 | 3 |
| Severe | 6 | 12 | 1 |
| Extremely severe | 7 | 3 | < 1 |

Table S18 Correlates of the severity of psychotic symptoms experienced in the past week

|  | Severity of psychotic symptoms in the past week |  |
| --- | --- | --- |
|  | OR (95% CI) | P value |
| **Time invariant (baseline) variables** |  |  |
| Family history of psychosis | 3.03 (1.41 – 6.50) | 0.005 |
| Male | 0.89 (0.47 – 1.70) | 0.726 |
| Age | 0.97 (0.93 – 1.00) | 0.067 |
| Years of schooling^a^ | 1.07 (0.89 – 1.28) | 0.474 |
| Unemployed | 1.26 (0.67 – 2.37) | 0.477 |
| Income^b^ |  |  |
| < 400 |  |  |
| 400-799 | 1.14 (0.39 – 3.32) | 0.813 |
| 800-1199 | 0.68 (0.21 – 2.19) | 0.518 |
| 1200+ | 0.78 (0.23 – 2.57) | 0.677 |
| Age of first methamphetamine use | 0.99 (0.96 – 1.03) | 0.622 |
| Duration of use | 0.98 (0.94 – 1.01) | 0.151 |
| **Past week drug use (time-varying)^c^** |  |  |
| Any methamphetamine use | 3.67 (1.90 – 7.10) | < 0.001 |
| Days of methamphetamine use | 1.25 (1.15 – 1.35) | < 0.001 |
| Any cannabis use | 1.58 (0.98 – 2.57) | 0.062 |
| Days of cannabis use | 1.08 (0.98 – 1.20) | 0.117 |
| Any alcohol use | 1.09 (0.80 – 1.49) | 0.572 |
| Days of alcohol use | 0.99 (0.90 – 1.10) | 0.901 |
| Any tobacco use | 1.01 (0.57 – 1.78) | 0.968 |
| Days of tobacco use | 0.98 (0.90 – 1.08) | 0.740 |
| Any other drug use^d^ | 1.64 (1.06 – 2.55) | 0.027 |

^a^AU$ net per fortnight

^b^12 years of schooling reflects completion of high school

^c^ Based on time-varying data for each week

^d^Use of any other drug class (cocaine, ecstasy, hallucinogens, inhalants heroin or other opioids)

Table S19 The odds of more severe psychotic symptoms in the past week by days of methamphetamine use in the past week stratified by a family history of psychosis

|  | Odds ratio (OR) of more severe psychotic symptoms in the past week | |
| --- | --- | --- |
|  | OR (95% CI) | P value |
| Unadjusted analysis: |  |  |
| No family history of psychosis | 1.25 (1.14 – 1.37) | < 0.001 |
| Family history of psychosis | 1.20 (1.01 – 1.42) | 0.034 |
| Adjusted analysis:^ |  |  |
| No family history of psychosis | 1.25 (1.14 – 1.37) | < 0.001 |
| Family history of psychosis | 1.25 (1.05 – 1.49) | 0.011 |

^Adjusted for any cannabis use in the past week, days of cannabis use in the past week, other drug use in the past week, days of tobacco use in the past week, age and years of schooling

Table S20 Interaction effect for family history of psychosis and days of methamphetamine use in the past week in predicting the severity of psychotic symptoms in the past week

|  | Days of methamphetamine use in the past week | | Family history of psychosis | | Interaction | |
| --- | --- | --- | --- | --- | --- | --- |
|  | OR (95% CI) | P value | OR (95% CI) | P value | OR (95% CI) | P value |
| Unadjusted analysis | 1.25 (1.14 – 1.36) | < 0.001 | 3.07 (0.77 – 12.25) | 0.112 | 0.99 (0.80 – 1.22) | 0.898 |
| Adjusted analysis^ | 1.24 (1.13 – 1.35) | < 0.001 | 2.51 (0.65 – 9.72) | 0.182 | 1.00 (0.81 – 1.23) | 0.989 |

^Adjusted for any cannabis use in the past week, days of cannabis use in the past week, other drug use in the past week, days of tobacco use in the past week, age and years of schooling

# References

**[1]** Hughes RA, Heron J, Sterne JAC, Tilling K. Accounting for missing data in statistical analyses: multiple imputation is not always the answer. *International Journal of Epidemiology* 2019;48:1294-304

**[2]** Pedersen AB, Mikkelsen EM, Cronin-Fenton D, Kristensen NR, Pham TM, Pedersen L, Petersen I. Missing data and multiple imputation in clinical epidemiological research. *Clin Epidemiol* 2017;9:157-66

**[3]** White IR, Royston P, Wood AM. Multiple imputation using chained equations: Issues and guidance for practice. *Statistics in Medicine* 2011;30:377-99

**[4]** Huque MH, Carlin JB, Simpson JA, Lee KJ. A comparison of multiple imputation methods for missing data in longitudinal studies. *BMC Medical Research Methodology* 2018;18:168

**[5]** De Silva AP, Moreno-Betancur M, De Livera AM, Lee KJ, Simpson JA. A comparison of multiple imputation methods for handling missing values in longitudinal data in the presence of a time-varying covariate with a non-linear association with time: a simulation study. *BMC Med Res Methodol* 2017;17:114

**[6]** Shah AD, Bartlett JW, Carpenter J, Nicholas O, Hemingway H. Comparison of random forest and parametric imputation models for imputing missing data using MICE: a CALIBER study. *Am J Epidemiol* 2014;179:764-74

**[7]** Graham JW, Olchowski AE, Gilreath TD. How many imputations are really needed? Some practical clarifications of multiple imputation theory. *Prevention Science* 2007;8:206-13

**[8]** Knol MJ, van der Tweel I, Grobbee DE, Numans ME, Geerlings MI. Estimating interaction on an additive scale between continuous determinants in a logistic regression model. *Int J Epidemiol* 2007;36:1111-8
